# Radioactivity and the environment: technical approaches to understand the role of arbuscular mycorrhizal plants in radionuclide bioaccumulation

**DOI:** 10.3389/fpls.2015.00580

**Published:** 2015-07-27

**Authors:** Helena S. Davies, Filipa Cox, Clare H. Robinson, Jon K. Pittman

**Affiliations:** ^1^Faculty of Life Sciences, The University of Manchester, Manchester, UK; ^2^School of Earth, Atmospheric and Environmental Sciences, The University of Manchester, Manchester, UK

**Keywords:** arbuscular mycorrhizal fungi, radioecology, radionuclide transport, radium, synchrotron X-ray fluorescence, uranium

## Abstract

Phytoaccumulation of radionuclides is of significant interest with regards to monitoring radionuclide build-up in food chains, developing methods for environmental bioremediation and for ecological management. There are many gaps in our understanding of the characteristics and mechanisms of plant radionuclide accumulation, including the importance of symbiotically-associated arbuscular mycorrhizal (AM) fungi. We first briefly review the evidence that demonstrates the ability of AM fungi to enhance the translocation of ^238^U into plant root tissues, and how fungal association may prevent further mobilization into shoot tissues. We then focus on approaches that should further advance our knowledge of AM fungi–plant radionuclide accumulation. Current research has mostly used artificial cultivation methods and we consider how more ecologically-relevant analysis might be performed. The use of synchrotron-based X-ray fluorescence imaging and absorption spectroscopy techniques to understand the mechanisms of radionuclide transfer from soil to plant via AM fungi is evaluated. Without such further knowledge, the behavior and mobilization of radionuclides cannot be accurately modeled and the potential risks cannot be accurately predicted.

## Introduction

Elevated levels of radioactive elements in the environment, resulting from industrial activities or geology, can be a significant problem to ecosystem well-being and may threaten human health, particularly if they build up in the food chain. Radionuclides occur naturally throughout the environment, with isotopes, such as ^238^U and ^226^Ra, originating from the Earth’s crust. Anthropogenic activities of mining and waste from nuclear energy generation can also enhance environmental radionuclide concentrations: for example, from accidental release or controlled discharge of radionuclides such as ^137^Cs, ^129^I, ^99^Tc, and Pu. Together these radionuclides can pose a radiological risk, in addition to inducing effects associated with chemotoxicity, such as oxidative stress (Saenen et al., 2013). Studies into the impacts of radionuclide concentration and contamination have focused primarily on the direct consequences to human health with less of a focus on the broader environmental and ecological consequences (Pentreath, 2009). Radioecology studies are beginning to redress this imbalance, particularly through understanding the behavior, mobility and partitioning of radionuclides into vegetation and the risks of food chain transfer.

A number of studies has examined the accumulation of non-essential radionuclides from the soil into various plant species in the context of radionuclide bioremediation (Dushenkov, 2003). In addition, investigations into the transfer of radionuclides into agricultural crops and edible plants are numerous because of the health risks associated with ingestion of radioactive materials (Pulhani et al., 2005; Laurette et al., 2012; Sokolik et al., 2014). However, notably fewer studies have analyzed the effects that associated arbuscular mycorrhizal (AM) fungi, have on radionuclide bioaccumulation into plants (Rufyikiri et al., 2002, 2003, 2004a,b; Chen et al., 2005a,b,c). Considering that 80–90% of plant species are symbiotically associated with AM fungi and their key position at the plant-soil interface (Smith and Read, 2008), this has been a significant oversight. AM fungi are obligate biotrophic symbionts, which form a (typically) mutualistic relationship with the roots of plant hosts, whereby in exchange for organic carbon from the host, the fungi assimilate and transfer phosphates and other nutrients into the plant (Govindarajulu et al., 2005; Smith and Read, 2008). Recent studies have, however, begun to appreciate the role that AM fungi also play in the accumulation of non-essential elements, including radionuclides, into plant tissues. Here we will briefly review the existing evidence about the role of AM fungal associations on plant radionuclide uptake obtained from earlier studies. We will then discuss the critical gaps in knowledge and this article will focus on the technical advances that we argue should allow a number of these gaps to be addressed. We consider the accumulation of U, and to a lesser extent Ra, as these are the major contributors to long-term radiological impact of spent fuel disposals as well as the main radioactive constituents of much naturally occurring radioactive material.

## Effects of AM Fungi on U Plant Bioaccumulation

Most plant species retain U in the roots with little shoot hyperaccumulation of this radionuclide (Dushenkov, 2003). Root sequestration of radionuclides may be a protective mechanism against chemotoxicity within shoot tissues and prevent photosynthetic inhibition (Weiersbye et al., 1999). For example, free UO_2_^2+^ is chemotoxic and can induce oxidative stress (Saenen et al., 2013). ^238^U is most readily bioaccumulated into plant roots as UO_2_^2+^, although uranyl carbonate complexes and UO_2_PO_4_^–^ can also be efficiently accumulated by plants (Günther et al., 2003; Mitchell et al., 2013). ^226^Ra is an important decay product of ^238^U and is often prevalent alongside ^238^U in natural environments that are rich in these radionuclides. ^226^Ra is also accumulated by plants as Ra^2+^, and like U it is retained in the roots in most plant species studied (Soudek et al., 2007; Mitchell et al., 2013). Several factors can directly affect chemical speciation and consequently radionuclide bioavailability for uptake into plants including soil type, organic matter concentration, Ca^2+^, K^+^, and PO_4_^3–^ concentrations, soil pH and microorganism abundance, including prevalence of AM fungi (Pulhani et al., 2005; Dupré de Boulois et al., 2008; Mitchell et al., 2013).

There is evidence that AM fungi can enhance the accumulation and/or tolerance of non-essential elements such as Pb and Cd, and radionuclides, including ^137^Cs, within AM fungal-associated roots but restrict accumulation into shoots, because of, in part, preferential hyphal accumulation and thus retention (Heggo et al., 1990; Joner and Leyval, 1997; Joner et al., 2000; Berreck and Haselwandter, 2001). As yet there have been no studies examining the effect of AM fungi on the plant accumulation of ^226^Ra or other U/Th decay chain radionuclides. However, a few studies have begun to investigate aspects of ^238^U translocation in AM fungi-associated plants. ^238^U uptake by extraradical mycelium of *Rhizophagus irregularis* (formerly *Glomus intraradices*) was investigated using a relatively simple, two-compartment *in vitro* growth culture to quantify the transfer of U from a hyphae-only outer compartment to an inner compartment containing a fungal-infected carrot root culture. This approach confirmed that *R. irregularis* was able to accumulate U in roots and indicated that U flux rates in the extraradical hyphae were significantly higher than in the roots (Rufyikiri et al., 2002, 2003). Subsequent experiments comparing mycorrhizal and non-mycorrhizal *Trifolium subterraneum* demonstrated that the inoculation of AM fungi can significantly decrease U shoot concentrations but only when grown in high concentrations of U (Rufyikiri et al., 2004b). Immobilization of ^238^U has also been observed within extra- and intra-radical hyphal structures (Weiersbye et al., 1999; Rufyikiri et al., 2004a; Chen et al., 2008), indicating that the fungi not only contribute to the accumulation of ^238^U, but also in limiting its translocation into above ground tissues. Similarly a decrease in root-to-stem ^238^U concentration was observed in *R. irregularis*-infected barley plants with an overall increase in root uptake of U (Chen et al., 2005b,c). *Medicago truncatula* associated with *R. irregularis* also demonstrated these effects (Chen et al., 2005a).

U is a non-essential element for fungi and plants, and the biological interactions of this element at the cellular level, including how it is transported and partitioned into the fungal and root tissues, are poorly understood. Correlations between ^238^U and phosphate uptake have been observed leading to the suggestion that the formation of stable U-phosphate complexes is the main mechanism behind ^238^U fungal uptake and may contribute to root and fungal tissue immobilization of ^238^U (Rufyikiri et al., 2003, 2004a). Complexation of ^238^U (as UO_2_^2+^) to polyphosphate appears to limit shoot translocation (Rufyikiri et al., 2002). ^238^U and P interactions are unlikely to be concentration dependent as increasing P fertilizer concentration on mycorrhizal-associated barley plants does not clearly correlate to ^238^U accumulation (Chen et al., 2005b; Dupré de Boulois et al., 2008). However, it is unlikely that only uranyl-phosphate complexes are assimilated by AM fungal hyphae because at pH values where other U forms, including uranyl sulfates and uranyl carbonates, are predominant, U is still efficiently assimilated by AM fungi (Rufyikiri et al., 2002). Despite these few experiments clearly demonstrating ^238^U AM fungal uptake, there are still many gaps in our understanding of radionuclide transfer into AM fungi-associated plants, both mechanistically and in an ecological context, which have not been addressed since these earlier studies 10 years ago.

## Improved Analysis Through the Use of High-Resolution Spectroscopy

Identifying exactly where U and other long-lived radionuclides are sequestered within fungal and plant tissues at a cellular level, and determination of the speciation of these sequestered radionuclides, should allow many unknown aspects of radionuclide accumulation to be addressed. Such an approach requires the ability to detect these elements at low concentrations and to high resolution. Synchrotron-based X-ray fluorescence (XRF) imaging and X-ray absorption spectroscopy (XAS) techniques including methods such as X-ray absorption near edge spectroscopy (XANES) have significantly advanced the ability to accurately quantify the distribution and determine the speciation of metals and metalloids within tissues (Punshon et al., 2009; Donner et al., 2012). XRF provides spatially resolved elemental mapping data while XAS methods, including XANES and extended X-ray absorption fine structure (EXAFS) spectroscopy, can provide speciation information including the oxidation state and the particular element complexes that are formed within tissue. These methods can similarly be used for the analysis of radionuclide-containing samples. For example, synchrotron EXAFS and laser-induced fluorescence spectroscopy were used to identify the preferred ^238^U species assimilated from soil pore water into the roots of *Lupinus* plants (Günther et al., 2003). U accumulated predominantly as UO_2_^2+^ and remained as U(VI) throughout the plant regardless of tissue localization. Furthermore, U was bound as U(VI)-phosphate complexes within the tissue. In an analogous study, use of EXAFS and XANES found that complexation of U with phosphate inhibited the translocation of U from roots to leaves (Laurette et al., 2012). These studies did not consider AM fungi, and as yet, no synchrotron-based U analysis experiments have been performed that examine plant tissue that is associated with AM fungi. However, more recent experiments studying the accumulation of the toxic metal Cd have attempted to use synchrotron analysis to differentiate the partitioning of elements between plant root cells and AM fungal cells.

**FIGURE 1:**
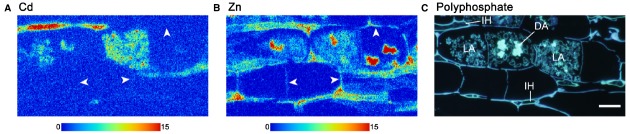
**Synchrotron radiation microscale X-ray fluorescence (μ-XRF) imaging of Cd (A) and Zn (B) within ***Lotus japonicus*** and ***Rhizophagus irregularis*** mycorrhizal roots.** The AM fungi-associated plants were grown in a multi-compartment growth system to separate the plant roots and fungal spores from fungal hyphae by a nylon mesh so that only the fungal hyphae were initially exposed to Cd-contaminated soil. Tissue sample was prepared by high-pressure freezing. Polyphosphate staining of the serial section by 4′, 6-diamidino-2-phenylindole fluorescence staining is shown in **(C)**. Color scale bars indicate minimum and maximum X-ray fluorescence counts. Arrowheads in **(A)** and **(B)** indicate plant host cell walls. In **(C)**, DA, dead arbuscule; IH, intercellular hypha; LA, live arbuscule. Reproduced from Nayuki et al. (2014) with permission.

The challenge with this approach is the ability to produce and preserve AM fungal-root tissue samples of suitable quality for microscopy or synchrotron analysis (Barrea et al., 2005; Nayuki et al., 2014). Clarity of intracellular structures can easily be lost and tissues can be completely degraded during sample preparation and during analysis (Weiersbye et al., 1999; Barrea et al., 2005; Nayuki et al., 2014). Digestion and subsequent homogenization of plant tissues can avoid these problems, but prevent chemical speciation mapping from being possible. Improved sample preparation for synchrotron imaging was therefore needed. Nayuki et al. (2014) compared two preparation methods for the production of resin-embedded extraradical hyphae and mycorrhizal roots for energy dispersive spectroscopy (EDS)-SEM and synchrotron microbeam-XRF (μ-XRF) in order to determine the localization of Cd and Zn at a cellular level. High pressure frozen, freeze-substituted and resin-embedded mycorrhizal root sections of *Lotus japonicus* and *R. irregularis* revealed an extremely well preserved ultrastructure under SEM (Nayuki et al., 2014). Serial sections of the resin embedded samples were able to give detailed Cd mapping by μ-XRF and demonstrated that much of the Cd was retained in fungal structures (Figure 1A), while the essential element Zn could be observed in plant and fungal structure (Figure 1B). These data in Figure 1 clearly show that individual elements can be distinguished between plant and fungal tissues. Furthermore, use of a different AM fungal-plant combination, *Allium cepa* and *Gigaspora margarita* allowed determination of a positive correlation between Cd and P distribution (Nayuki et al., 2014).

This study by Nayuki et al. (2014) strongly demonstrates the potential of synchrotron-based imaging for the analysis of other elements such as U in AM fungal-plant tissues. Replicating such methods for mycorrhizal plants exposed to ^238^U would be useful to determine how much of the radionuclide is retained within fungal cells and how much is translocated into roots and shoots. It could also allow for chemical speciation changes to be tracked along the biological translocation pathway. Furthermore, EXAFS analysis could determine the local and extended chemical coordination of ^238^U to confirm oxidation state and reveal whether complexation to phosphate does occur. However, interpretation of similar ^226^Ra data might be highly problematic due to the close chemistry of Ra with Ba (Vandenhove et al., 2005). A further challenge is that soil particles adhered to roots or mycelium can completely destroy the integrity of the samples and prevent fine slicing of resin embedded samples. Thus, Nayuki et al. (2014) used a hydroponic and nutrient agar growth medium with the compromise of using soil as the source of Cd. Günther et al. (2003) compromised by growing root samples in U-doped soil pore water, thereby removing the problem of soil particulates on samples. However, this system might exclude U sources that become indirectly bioavailable from AM fungal breakdown of organic matter, leading to underestimation of U uptake. Nevertheless, these studies suggest that considerable insight into the role of AM fungi on radionuclide transfer will be gained through the use of these synchrotron-based analytical techniques.

## A Novel Approach to AM Fungal–Plant Radionuclide Accumulation Analyses: A Need to Determine Environmental Relevance

Many of the available studies on U fungal-plant transfer lack ecological relevance. Use of hydroponics or tissue culture systems (Rufyikiri et al., 2002, 2003) enable specific variables to be determined, but do not allow factors such as soil adsorption of radionuclides to be represented, whilst their applicability to ecological systems is unknown. In contrast, use of natural sources of ^238^U and soil from actual contaminated sites are more likely to correspond to what would actually occur in the field, particularly if using environmentally relevant concentrations of radionuclides in a form that has reached steady-state. Furthermore, the use of *R. irregularis* in monoxenic cultures, often twinned with a model plant species, has dominated investigations. Despite the obvious advantages of using model species, this undoubtedly restricts the environmental relevance of the conclusions. Not only is it extremely rare for a plant to be colonized by a single AM fungal species, but also AM fungal colonization of plant species is not random and can display a marked degree of selectivity (Helgason et al., 2002; Ji et al., 2010). In addition, variance between the diversity and extent of AM fungal species colonization on plant roots has been shown to affect P uptake levels and plant biomass production (Helgason et al., 2002). As P uptake efficiency has been linked to ^238^U accumulation, an inappropriately matched fungal/plant species combination could indirectly result in under- or over-estimation of radionuclide translocation. It would therefore be of value for ecological surveys of radionuclide contaminated sites to determine AM fungal species diversity and quantify the extent of colonization on the roots of plants studied to help alleviate some of these problems. This would boost AM fungal diversity knowledge in general (Fitter et al., 2011) and would provide closer matching of likely plant/fungal partners in future experiments. Not only would this allow more accurate experimental design, but could also be a resource in the better use of plants for radionuclide phytoremediation.

There are many difficulties in working with AM fungi, which may account for why so many studies have either omitted the consideration of fungi completely or used plant species (e.g., within the Brassicaceae) which do not form AM fungal symbiosis (Soudek et al., 2007). Moreover the general difficulty in implementing control samples within AM fungi-based experiments can be highly significant. Spore contamination can result in root colonization of an unwanted species and can therefore cause AM fungi population shifts in favor of the contaminant as opposed to the inoculants of interest. Soil has often been sterilized in order to remove unwanted biota, however, sterilization techniques can alter the physical and chemical properties of soil such as by causing severe nutrient leaching and thus creating a phytotoxic growing environment (Johnson et al., 2001). Another challenge is that AM fungi cannot be cultured without a plant symbiont and consequently this can make it difficult to separate the contributions made by fungi to that of the plant. Similarly, plants and fungi cannot be grown in media lacking essential nutrients, such as phosphate, which might complicate the interpretation of U bioaccumulation.

**FIGURE 2:**
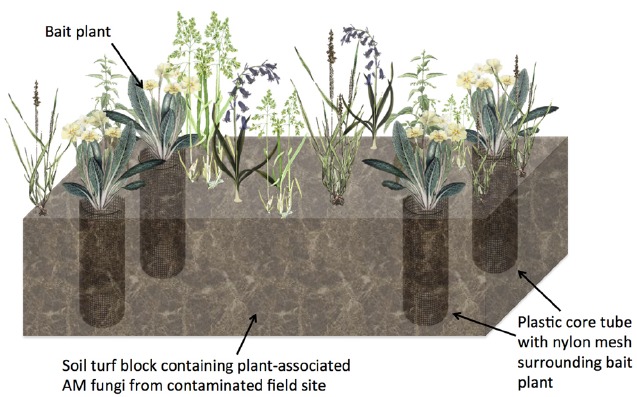
**Diagrammatic representation of an intact turf based experiment to examine the effects of AM fungal colonization on non-essential element or radionuclide accumulation into plant tissue.** A soil turf block containing natural vegetation and associated AM fungi from a field site such as a natural ^238^U-rich soil site is removed and plastic core tubes containing an added “bait” plant in soil to attract fungal hyphae are added. Part of the tube is cut away and covered by nylon mesh to prevent root growth outside the core but allows hyphae to penetrate the core and infect the bait plant root. Cores may be rotated to sever fungal hyphae connections in order to differentiate between fungal associated and non-fungal associated bait plants.

To improve the ecological relevance of these studies, more versatile methodologies are needed. For example, a method for the study of AM fungal function in grassland systems (Johnson et al., 2001) could be used for studying radionuclide accumulation. Such a mesocosm experiment uses a mesh-lined core system to control mycorrhizal association cleanly (Figure 2). In brief, rectangular soil/turf plots (so-called “monoliths”) are removed intact (with existing vegetation on the monolith undisturbed) from the environment of interest and cores are extracted and lined with plastic pipe sections cut with large windows that are covered with a 35 μm pore-sized nylon mesh. After adding sterilized sand or soil into the core, so called “bait” plants are added (Figure 2). The concept is that the undisturbed fine mesh allows AM fungal hyphae to pass through, but is too fine to allow penetration of plant roots, whilst rotation of the mesh causes AM fungal hyphal severing (Johnson et al., 2001). With the monolith providing the source of AM fungi, this experimental setup allows comparison of control plants without fungal association and mycorrhizal root conditions, without causing drastic changes to the physical-chemical properties of the soil. Furthermore, by extracting soils from radionuclide contaminated environments, natural, steady-state sources of the radionuclide would be used.

## Perspectives and Implications

The near-surface behavior of radionuclides is critical in the assessment of contaminated land and the consequences of radioactive accidents at sites around the world. Contaminated sites require long (multi-decades) and expensive (billions of dollars) remediation programs (IAEA, 2006). The nuclear industry in many parts of the world has resulted in a nuclear legacy that is one of the most demanding scientific and environmental challenges for the next century (Greenberg, 2013). For example, there may be the potential for return of radionuclides to the near-surface from high activity wastes in a geological disposal facility. Therefore it is essential to understand fully how the biogeochemical behavior and ecological transfer of radionuclides in the near-surface environment may be affected and controlled by biological processes, such as the action of fungi and plants. Such knowledge will allow us to understand the impacts of radioactivity on individual plants and on whole ecosystems more clearly, and have additional implications on the design characteristics of geological disposal facilities. Furthermore, an understanding of the characteristics of fungal-plant radionuclide accumulation will provide an evaluation of the effectiveness of using such material for radionuclide bioremediation.

The evidence to date demonstrates that the root systems of plants can mediate the uptake and translocation of radionuclides from the soil into shoots, with the potential for further transfer along a food chain. However, it has become clear that modeling this transfer pathway as a two-step process, from soil-to-root, is unrealistic compared with actual pathways, as AM fungi must also be considered. Many gaps in knowledge still remain, especially concerning AM fungal contributions to ^232^Th and ^226^Ra accumulation, and a lack of experimental evidence behind the mechanisms of ^238^U, ^232^Th, and ^226^Ra soil-fungi–plant transfer and tissue partitioning. We propose that high resolution imaging and quantification tools such as synchrotron X-ray spectroscopy will be vital. Another gap in knowledge is whether plant and AM fungi species diversity is affected by radionuclides, and whether these organisms exhibit physiological and/or genetic adaptation to the radionuclide burden. Most importantly from an ecological perspective, experimental analyses need to replicate better the real environment rather than emphasizing artificial growth systems to allow more accurate understanding of the environmental impacts of radionuclides. Without such knowledge, the behavior and mobilization of these radionuclides cannot be accurately modeled and the potential risks cannot be accurately predicted.

### Conflict of Interest Statement

The authors declare that the research was conducted in the absence of any commercial or financial relationships that could be construed as a potential conflict of interest.
